# Design and Characterization of Surface Acoustic Wave-Based Wireless and Passive Temperature Sensing System

**DOI:** 10.3390/mi15040544

**Published:** 2024-04-18

**Authors:** Zhixin Zhou, Hui Wang, Liang Lou

**Affiliations:** 1 School of Microelectronics, Shanghai University, Shanghai 201800, China; zzx1999@shu.edu.cn (Z.Z.); wanghui1996@shu.edu.cn (H.W.); 2 The Shanghai Industrial µTechnology Research Institute, Shanghai 201899, China

**Keywords:** surface acoustic wave resonator, aluminum nitride, temperature sensor, wireless sensing system

## Abstract

The surface acoustic wave (SAW) temperature sensor has received significant attention due to its wirelessly powered, battery-free, and chipless capabilities. This paper proposes a wireless sensing system comprising a one-port SAW resonator, helix antenna, and transceiver circuit. The SAW resonator used in this system is based on aluminum nitride (AlN) thin film, which exhibits high velocity and excellent piezoelectric properties. Simulations and experiments were conducted to investigate the performance of the designed SAW resonator. A helix antenna was also designed using finite element simulation to facilitate signal transmission between the SAW temperature sensor and the transceiver. An impedance-matching network was introduced between the helix antenna and the SAW resonator to optimize signal transmission. When the wireless SAW temperature sensor was placed within a certain distance of the mother antenna, the reflection peak of the SAW resonator was observed in the spectrum of the return signal. The frequency of the echo signal increased almost linearly as the temperature increased during the temperature tests. The fitted temperature coefficient of frequency (TCF) was −31.34 ppm/°C, indicating that the wireless temperature sensing system has high-temperature sensitivity.

## 1. Introduction

With the development of the intelligent era, the demand for electricity in society is increasing. To cope with this trend, it is necessary to ensure the stable operation of the power system in addition to the continuous innovation and reform of the power system [1]. High-voltage switchgear is an important component of power equipment with a complex internal structure, operating in a closed environment and under high voltage and current conditions. When the electrical resistance of the switch contacts ages or the contact resistance becomes too high, it can lead to a continuous increase in temperature, ultimately resulting in accidents such as fires and power outages [2]. To prevent such incidents, real-time temperature monitoring is required in high-voltage switchgear. Existing temperature sensors used to detect heating faults in switchgear include infrared thermometers, fiber optic temperature sensors, and surface acoustic wave (SAW) sensors [3]. Infrared thermometers require regular manual inspection and cannot accurately measure the temperature inside the switchgear due to the sealed structure of the switchgear. Fiber optic temperature sensors have strong anti-interference capabilities but require specific installation locations, which may be challenging given the limited space within the switchgear and may hinder their effectiveness. Passive wireless temperature sensors, on the other hand, can effectively overcome these challenges and have become a popular focus of current research [4]. SAW temperature sensors, with their advantages of small size, high sensitivity, easy integration, and the ability to be passive and wireless, have been widely applied in various fields, such as gas [5,6], humidity [7,8], and pressure [9,10]. They represent a highly promising passive wireless monitoring technology with extensive application prospects [11].

Over the past few decades, numerous wireless SAW temperature sensors have been invented and reported. In 2000, M. Binhack et al. successfully used SAW sensors to achieve the passive wireless sensing of external physical parameters [12]. Subsequently, Keekeun Lee et al. developed a 440 MHz delay-line SAW pressure–temperature sensor, which computed temperature variations by detecting phase changes in the signals, enabling a temperature measurement range of 20–200 °C with a sensitivity of 10°/°C [13]. V. Kalinin et al. designed a high-speed reader capable of measuring a differential wireless SAW sensor, analyzing cases of mutual interference between the return signals of two sensors [14]. TranSense Technologies, a company based in the UK, specializes in the design of SAW sensors. They have developed three SAW resonators in three directions on the quartz substrate, enabling the simultaneous detection of temperature and pressure changes. They also designed an antenna and reader circuit for this sensor, analyzing the matching between the SAW resonators and the antenna, ultimately achieving the passive wireless monitoring of pressure and temperature changes [15]. Similarly, the French company SenSeOR specializes in SAW resonators for wireless sensing. In 2006, the company achieved the passive wireless monitoring of ship engine temperatures using SAW sensors, with a test distance of up to ten meters. The company subsequently explored various applications of SAW sensors in other fields [16,17,18,19], including passive wireless temperature monitoring devices for high-voltage switchgear. Moreover, internationally renowned companies such as BaumerIdentGmbH, Siemens in Germany, Sengenuity in the United States, and CarinthianTechResearch (CTR) in Australia have carried out extensive research into passive wireless temperature measurement technology based on SAW sensors. Many of their products have been put into practical use, demonstrating the promise of SAW passive wireless temperature sensors.

Commonly used SAW piezoelectric materials such as quartz, lithium niobate, and lithium tantalate are not suitable for high-temperature applications above 500 °C due to phase transition, decomposition, and high acoustic propagation losses [20,21]. Langasite (LGS) can maintain stability above 1000 °C in an atmospheric environment and is considered a strong competitor as a harsh environment SAW piezoelectric material [22]. However, LGS has drawbacks such as low sound velocity (2500–3000 m/s), decreasing resistivity with temperature, and significant surface roughness at high temperatures [23]. Studies have shown that the temperature sensitivity of SAW devices increases with frequency, but the acoustic propagation losses of LGS also increase with frequency, limiting the potential for further sensitivity enhancement using this approach [24]. Another candidate material for high-temperature SAW applications is aluminum nitride (AlN). AlN has high thermal and chemical stability above 1000 °C and a high acoustic velocity of 5500 m/s, making it compatible with high-frequency applications [25,26]. In addition, an effective way to improve the temperature coefficient of frequency (TCF) is to choose appropriate layer designs without the need to increase the frequency [27]. As a piezoelectric thin film material, AlN is easier to achieve compared to piezoelectric crystal materials such as LGS. Therefore, AlN has great potential and advantages for high-temperature SAW applications.

In recent years, researchers have conducted extensive studies on AlN-based SAW temperature sensors. Alexandra Nicoloiu et al. investigated the performance of high-frequency AlN SAW temperature sensors based on (111) Si and glass substrates, achieving high sensitivities of −490 kHz/°C and −306 kHz/°C, respectively [28]. Lin Shu et al. directly deposited AlN thin films on TC4 alloy and processed them into SAW temperature sensors to explore the feasibility of fabricating SAW sensors directly on the tested alloy [29]. Jérémy Streque et al. optimized the structure of the AlN/sapphire temperature sensor by introducing detuning between the interdigital transducer (IDT) and reflector to obtain a high-Q SAW temperature sensor and investigated the variation in Q with temperature [30]. Leonardo Lamanna et al. fabricated AlN SAW resonators on a flexible polyethylene naphthalate (PEN) substrate and successfully excited Rayleigh, Love, and Lamb wave modes with TCFs of −149 ppm/°C, −109 ppm/°C and −53 ppm/°C, respectively [31]. A. Müller et al. investigated the influence of electrode thickness on the temperature performance of AlN SAW resonators and found that the thicker the electrode, the higher the TCF of the device [32]. In summary, researchers have conducted extensive studies on the application of AlN as a temperature sensor, demonstrating its promising prospects. However, most of these studies have been based on cable or network analyzer (VNA) tests, and the performance of wireless temperature sensors based on AlN is not yet clearly understood.

This paper presents a wireless sensing system comprising a one-port SAW resonator, an antenna, and a transceiver circuit. The SAW resonator in this system is based on an AlN thin film operating at 502 MHz. To enable wireless communication between the sensor and the transceiver, a helical antenna is selected as the carrier for the temperature sensor. Finite element simulations are employed to optimize the dimensional parameters of the SAW resonator and the helical antenna. After fabrication, basic electrical tests are conducted to validate the rationality of the device’s design. Subsequently, temperature measurement experiments are carried out upon completion of the sensor assembly.

## 2. Materials and Methods

### 2.1. Basic Principles of The Wireless SAW Temperature Sensing System

**Figure 1:**
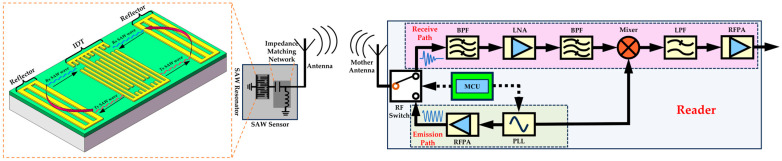
Schematic diagram of the constructed wireless temperature measurement system.

To achieve wireless passive SAW temperature sensors, the construction of the reader circuit is crucial. The SAW resonator captures electromagnetic excitation signals using an antenna, stores energy and transmits sensing signals through the same antenna. Therefore, the reader circuit should include both the emission path for generating electromagnetic excitation signals and the receive path for capturing and processing sensor return signals. The schematic diagram of the wireless temperature measurement system constructed in this study is shown in Figure 1. The microcontroller unit (MCU) does not generate or process signals but controls the operating state of the radio frequency switch (a single-pole double-throw switch) and the phase-locked loop (PLL). The PLL is a circuit that can generate the required frequency signal after receiving the reference signal from the MCU. In this system, the PLL is used to generate the interrogation signal for the emission path and the local signal required by the mixer. The frequencies of both signals match the resonance frequency of the SAW resonator. As the emission path and the reception path share a common mother antenna, a single-pole double-throw switch is needed to control the connection between the mother antenna, the emission path, and the receive path.

A complete temperature measurement cycle is described as follows:

(1) Firstly, the MCU controls the PLL to generate a continuous sinusoidal interrogation signal at the required frequency. This signal is then amplified by the radio frequency power amplifier (RFPA). The MCU also controls the single-pole double-throw switch to disconnect the mother antenna from the receive path and connect it to the emission path. At this point, the mother antenna is in a continuous transmission state, and the interrogation signal is radiated into the air through the mother antenna.
(2) The sensor antenna captures the electromagnetic interrogation signal and transmits it to the IDT of the SAW resonator. An impedance-matching network is used to match the impedances of the antenna and the resonator, allowing for maximum energy transfer between the two. The IDT converts the voltage signal into SAWs propagating in both directions. When the SAWs encounter the reflector of the Bragg structure, they reflect and form a standing wave. As the interrogation signal is continuous, the resonator remains in a sustained resonance state. Temperature fluctuations cause variations in parameters such as the width of the IDT and the elastic constants of AlN during the resonance of the resonator, leading to changes in its resonance frequency [33].
(3) Once the transceiver has emitted the interrogation signal continuously for a period, the MCU controls the single-pole double-throw switch. This connects the mother antenna to the receive path while disconnecting it from the emission path. As a result, the mother antenna is in the receiving phase and stops emitting interrogation signals into the air. At this point, if the sensor antenna fails to receive the interrogation signal, the resonator releases the accumulated energy. The SAWs are converted into an electrical signal by the IDT, which is then transmitted into the air via the sensor antenna. The energy stored in the resonator is finite and subject to losses, causing the residual amplitude of the SAW to gradually decrease until it ceases entirely. This results in a transient signal of limited duration and gradually decaying waveform that characterizes the return signal of the wireless passive SAW sensor.
(4) The return signal is received by the mother antenna, which remains connected to the receiving path at this point, allowing the echo signal to enter the receiving path for processing. The signal then passes through a bandpass filter (BPF) to eliminate noise from other frequency bands. Next, the signal is amplified using a low-noise amplifier (LNA) and then passed through another bandpass filter (BPF) to reduce noise. During the sampling of the echo signal, to accurately reconstruct the original signal, the sampling frequency needs to be more than twice the frequency of the echo signal. To reduce this requirement, a mixer is used to shift the echo signal’s frequency. If the input of the mixer has a local signal frequency of *w*_1_ and a return signal frequency of *w*_2_, the output signal is a mixed signal possessing two frequencies, the ultra-high frequency *w*_1_ *+ w*_2_, and the intermediate frequency *w*_1−_*w*_2_. Subsequently, a low-pass filter (LPF) is used to eliminate the high-frequency signal, leaving only the intermediate-frequency signal. Finally, the signal is amplified again using RFPA before being output for collection and analysis. In future research, we aim to explore the direct use of an MCU for signal acquisition and analysis to obtain real-time temperature information.

### 2.2. Simulation of the SAW Resonator

**Figure 2:**
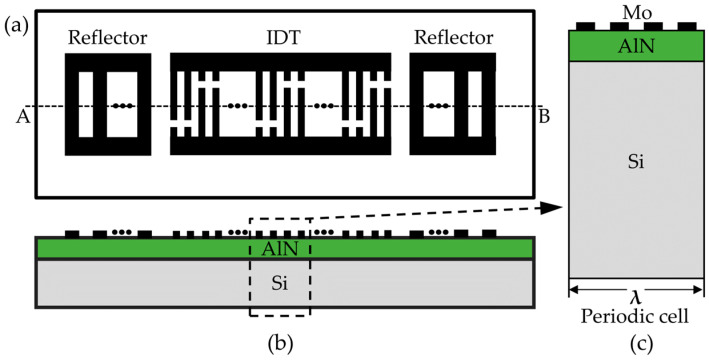
Schematic of the top view (**a**), the cross-sectional view at (A-B) (**b**), and the modeling periodic cell (**c**) of the one-port SAW resonator.

Figure 2 illustrates the design of a single-port SAW resonator, which consists of 64 pairs of IDTs in the middle and 75 pairs of short-circuit reflectors on each side. To enhance insertion loss, we reduced lateral parasites and achieved a higher Q-factor, while split electrodes and dummy finger electrodes were also used [34,35]. The width and spacing of the IDT are both λ/8, equivalent to 1.2 μm, resulting in a wavelength of 9.6 μm. The reflector width and spacing are λ/4, i.e., 2.4 μm. The aperture size *W* is 54λ. Mo was chosen as the top electrode material due to its high acoustic impedance, low density, and excellent high-temperature performance [36]. Although a thicker electrode can increase the temperature sensitivity of the SAW resonator, it may degrade performance due to mass-loading effects [32]. Therefore, the electrode thickness was set at 200 nm to achieve a balance between these factors. The Q-factor and electromechanical coupling coefficient *k*^2^ of the SAW resonator are closely linked to the thickness of the piezoelectric layer [37]. To balance performance and manufacturing capabilities, the thickness of the piezoelectric layer made of AlN was set at 1 μm. The substrate used in this study is polycrystalline silicon, with a thickness of 725 μm. The performance of the designed SAW resonator was predicted using the finite element simulation software COMSOL Multiphysics 6.0. Since the IDT is periodically repeated to reduce computational load, only one periodic unit was modeled with periodic boundary conditions applied on both sides for the simulation. As SAW propagates only on the solid surface with a depth of 1 to 2 wavelengths, the silicon substrate thickness was simplified to 2.5λ. The established 2D simulation model is depicted in Figure 2c. Table 1 presents the physical parameters of the materials used in the simulation. It also displays the first-order temperature coefficients of the material parameters used to simulate the temperature–frequency characteristics of the designed SAW resonator.

### 2.3. Fabrication of the SAW Resonator

**Figure 3:**
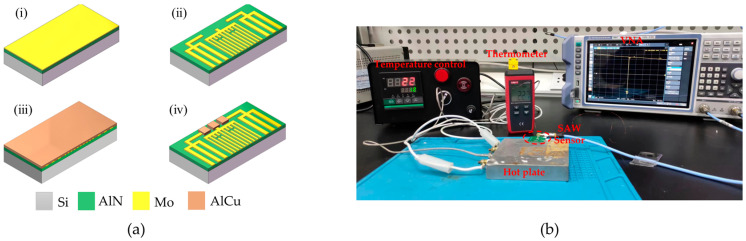
(**a**) SAW fabrication process flow diagram: (**i**) Deposition of AlN and Mo; (**ii**) Mo pattern; (**iii**) deposition of AlCu; and (**iv**) AlCu pattern; (**b**) wired temperature test platform of the SAW resonator.

The designed SAW resonator was fabricated on an 8-inch polycrystalline silicon wafer using standard photolithography processes. Figure 3a illustrates a three-dimensional cross-sectional schematic of the process flow for the designed SAW resonator. The device depicted in the figure represents half of the SAW resonator cut along the AB line in Figure 2a. After ultrasonic cleaning of the wafer, AlN with a thickness of 1 μm and Mo with a thickness of 0.2 μm were deposited using magnetron sputtering. Subsequently, after photoresist exposure, reactive ion etching was employed to pattern the Mo electrodes. Following that, a 1 μm thick layer of aluminum copper (AlCu) was deposited on the surface. Dry etching was then used to form bonding pads after photoresist exposure, which allowed for better wire bonding with the printed circuit board (PCB).

### 2.4. Characterization of the SAW Resonator

After fabrication and dicing, the SAW resonator was affixed to a PCB using a thermally conductive adhesive. The SAW pads were connected to the PCB pads through gold wires. The opposite end of the PCB was fitted with an SMA connector, which was connected to a Vector Network Analyzer (VNA, ROHDE&SCHWARZ ZNL6, Munich, Germany) via a cable for preliminary electrical characterization of the SAW resonator at room temperature. The PCB was then bonded to a heating plate using a thermally conductive adhesive for device heating. The *S*_11_ parameter of the SAW resonator was measured using the VNA to determine the temperature dependence of the resonant frequency of the SAW resonator under wired testing conditions. The constructed wired test platform is shown in Figure 3b. The temperature varied from 30 °C to 150 °C in steps of 10 °C. During the testing process, due to the non-uniform heating of the heating plate surface, approximately 10 min was required to stabilize at each temperature point. Additionally, to ensure the reliability of the test data, a thermocouple temperature sensor (UniTrend Technology UT320A, Wu Xi, Jiangsu, China) probe was affixed to the SAW resonator on the PCB using a thermally conductive adhesive to accurately monitor the temperature of the resonator.

### 2.5. Preparation of the Helix Antenna

In this design, a helix antenna was used for signal transmission between the SAW temperature sensor and the reader, as the helix antenna has the advantages of simple structure, wide bandwidth, and circularly polarized radiation. The frequency of the antenna must match the resonance frequency of the SAW resonator. This ensures that the antenna can receive the interrogation signal effectively and stimulate the SAW resonator. This is because the frequency of the interrogation signal also matches the frequency of the SAW resonator. Furthermore, since the resonant frequency of the SAW resonator changes with temperature, the bandwidth of the helix antenna must cover the entire resonant frequency range of the SAW within the temperature measurement range.

**Figure 4:**
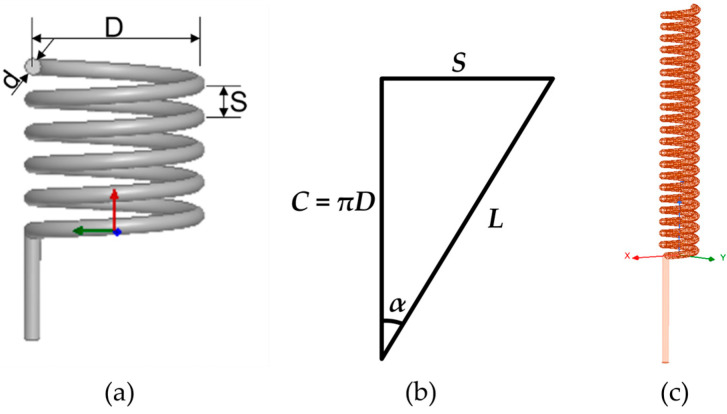
(**a**) Schematic diagram of the helix antenna; (**b**) relationship between space (*S*), helix diameter (*D*), angle of lift (α), and circle length (*L*); and (**c**) modeled helix antenna in HFSS.

Figure 4a illustrates the schematic diagram of the helix antenna, where *D* represents the diameter of the helix, *S* is the spacing between two helixes, *N* is the number of turns of the helix antenna, and *d* is the diameter of the conductor of the helix antenna. The helical antenna’s loop can unfold into a plane, forming a right triangle with perimeter *C*, loop length *L*, and helical space *S*, as shown in Figure 4b. In the figure, *α* represents the helix pitch angle, where a value of 90° corresponds to a straight-line antenna, and 0° corresponds to a circular antenna. The antenna’s operating mode is determined by the ratio of the helix diameter to the wavelength. For this study, the antenna operates at a frequency of around 500 MHz, and the wavelength can be calculated using the following formula:

(1) $$\lambda =\frac{c}{f}$$

where *c* is the speed of electromagnetic waves. When *D*/λ is less than 0.18, the helix antenna’s maximum radiation direction is within the plane perpendicular to the helix axis, known as the normal mode helix antenna. When *D*/*λ* is between 0.25 and 0.46, the antenna has maximum radiation along the axial direction, known as the axial mode helix antenna. As *D*/*λ* increases further, the radiation pattern transitions into a conical shape. The maximum radiation direction lies between the orientations perpendicular and parallel to the antenna axis, which is known as the conical mode helix antenna. Although the axial mode antenna offers higher gain, its larger size is a disadvantage. Conversely, the normal mode helix antenna has a compact structure, high radiation efficiency, and wide bandwidth, making it particularly well-suited for SAW sensor applications.

The AnsysEM21.2 High-Frequency Structure Simulator (HFSS), a finite element simulation software, was used to design and optimize the dimensions of the antenna. The HFSS Antenna Design Kit can automatically generate models based on the antenna’s dimensional parameters. The helix antenna used in this study was optimized based on a commercially available 433 MHz helix antenna. Figure 4b shows the model generated in HFSS. As the conductor diameter is fixed, adjustments to the antenna’s performance are achieved by varying the helical space, number of turns, and helical diameter. Simulation validation showed that, within a certain range, the frequency of the helical antenna increases as the helical space increases. However, when the space exceeds a certain threshold value, the frequency shows a negative correlation with the space. The number of helical turns and the helical diameter are inversely proportional to the frequency of the helical antenna. Specifically, an increase in the number of turns and helical diameter results in a decrease in the antenna’s frequency. Based on these principles, adjustments were made to the parameters of the helical antenna, as shown in Table 2. The antenna was constructed using copper wire in accordance with the specifications provided in Table 2.

### 2.6. Setup of the Wireless Temperature Test Platform

**Figure 5:**
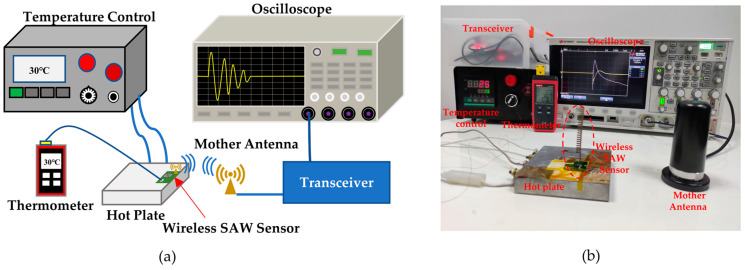
(**a**) Schematic diagram of the wireless temperature test platform; (**b**) self-built temperature test platform.

The SAW resonator and the helical antenna are connected via a PCB. An impedance-matching network is constructed using a capacitor and an inductor to facilitate optimal energy transfer between the two components. A temperature test platform, as depicted in Figure 5, was established to assess the wireless temperature sensing capabilities of the SAW sensor. This platform includes a temperature controller, a heating plate, a thermocouple, a temperature sensor, a transceiver with a mother antenna, and an oscilloscope (KEYSIGHT DSOX2014A, Santa Rosa, CA, USA) for signal observation. The SAW temperature sensor is attached to the heating plate for thermal activation using a thermally conductive adhesive. To enhance the real-time accuracy of the temperature of the SAW sensor, a thermocouple wire with a resolution of 0.1 °C was glued near the SAW resonator. During temperature measurements, to ensure stable temperature conditions for the SAW resonator, the system was allowed to remain at the desired temperature for ten minutes before data acquisition began.

## 3. Results and Discussion

**Figure 6:**
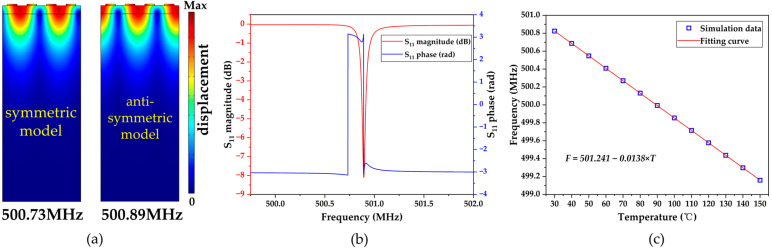
Simulation results of SAW resonator: (**a**) Rayleigh symmetry mode and anti-symmetry mode; (**b**) magnitude and phase of return loss *S*_11_; and (**c**) variation in the resonant frequency with temperature.

Figure 6a shows the simulated symmetric and anti-symmetric modes of the SAW resonator at frequencies of 500.73 MHz and 500.89 MHz, respectively. It can be observed from the figure that the SAW propagates through all the layers, indicating that the device’s performance is not solely dependent on the piezoelectric layer but is closely related to each layer. Figure 6b displays the simulated magnitude and phase diagram of the feedback coefficient *S*_11_ of the SAW resonator, revealing an *S*_11_ value of −8.1 dB at 500.89 MHz. The temperature–frequency relationship of the SAW resonator was also simulated, as shown in Figure 6c. It demonstrates that as the temperature increases, the frequency of the SAW resonator decreases linearly. This phenomenon is attributed to the following two factors: changes in the SAW wavelength caused by thermal expansion of the thin film and changes in physical parameters, such as elastic constants, resulting in a decrease in the speed of SAW. This relationship can be expressed as follows [30]:

(2) $$TCF=\frac{1}{f_{0}}\frac{\partial f}{\partial T}=\frac{1}{v_{p}}\frac{\partial v_{p}}{\partial T}-\frac{1}{\lambda }\frac{\partial \lambda }{\partial T}=\frac{1}{v_{p}}\frac{\partial v_{p}}{\partial T}-\alpha^{\prime }$$

where *f*_0_, *v_p_*, and *λ* are the resonant frequency, sound velocity, and wavelength of the SAW at the initial temperature, respectively. And αʹ is the effective thermal expansion coefficient (*TEC*) of the entire layered structure. In the equation, TCF is the temperature coefficient of frequency, which is commonly used to evaluate the temperature stability of devices and is defined as the relative rate of change in the device’s response frequency with temperature [39]. It can be calculated using the following formula:

(3) $$TCF=\frac{1}{T-T_{0}}\frac{f\left(T\right)-f\left(T_{0}\right)}{f\left(T_{0}\right)}\times 10^{6}$$

where *T*_0_ represents the initial temperature, *T* represents the final temperature, *f(T*_0_*)* is the center frequency of the SAW resonator at *T*_0_, and *f(T)* is the center frequency of the SAW resonator at *T*. The calculated simulation temperature sensitivity and TCF are −13.8 kHz/°C and −27.71 ppm/°C, respectively. Since only the first-order temperature coefficients of the material parameters are incorporated in the simulation model, the simulation results exhibit a first-order linear characteristic.

**Figure 7:**
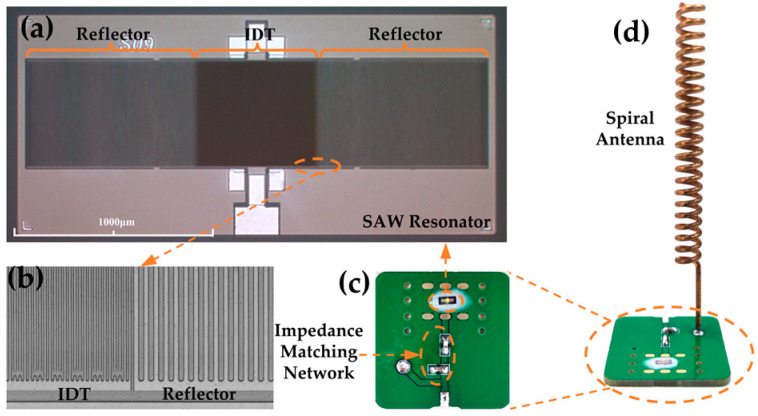
(**a**) Micro-view of the designed SAW resonator; (**b**) micro-view of the IDT and reflector; (**c**) view of the SAW sensor with impedance matching network; and (**d**) view of the wireless SAW temperature sensor.

**Figure 8:**
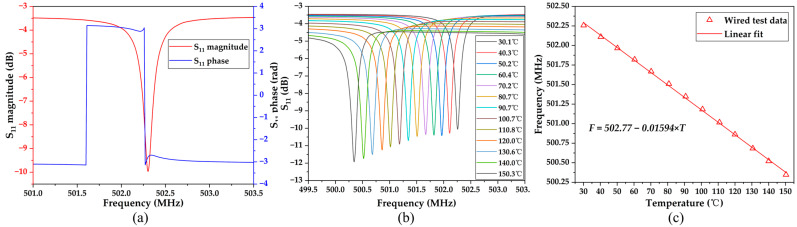
Wired measured results of the SAW resonator (**a**) magnitude and phase of return loss *S*_11_; (**b**) the *S*_11_ curve at different temperatures; and (**c**) the frequency of the SAW resonator as a function of temperature.

Figure 7a shows the microscopic image of the designed SAW resonator, while Figure 7b presents the 3D microscopic view of the IDT and reflector of the SAW resonator measured by a profilometer. The sidewalls of the IDT and reflectors are smooth and uninterrupted. Figure 8a shows the *S*_11_ curve of the tested SAW resonator, which shows a significant peak at 502.3 MHz with an amplitude of −9.97 dB. The slight discrepancy between the measured and simulated results may be due to incomplete alignment between the simulated and actual material parameters, process-induced variations in electrode thickness and width, and electrical connectivity issues. Furthermore, Figure 8b illustrates the *S*_11_ curves of the SAW resonator at different temperatures. It is observed that as the temperature increases, the resonant frequency of the SAW resonator continuously decreases from 502.26 MHz at 30.1 °C to 500.35 MHz at 150.3 °C, resulting in a total decrease of 1.91 MHz. Furthermore, by further extracting the frequency–temperature relationship of the SAW resonator from Figure 8b and performing linear fitting, the results are shown in Figure 8c. The calculated temperature sensitivity of the designed SAW resonator is −15.89 kHz/°C, demonstrating an excellent linearity of 0.99889. High linearity improves measurement accuracy, reliability, and precision, facilitates calibration, and is valuable in many applications [40]. Further calculations using Equation (2) provide a TCF of −31.63 ppm/°C for the SAW resonator, with some slight deviation from the simulation, possibly due to process variations and material parameter inaccuracies.

**Figure 9:**
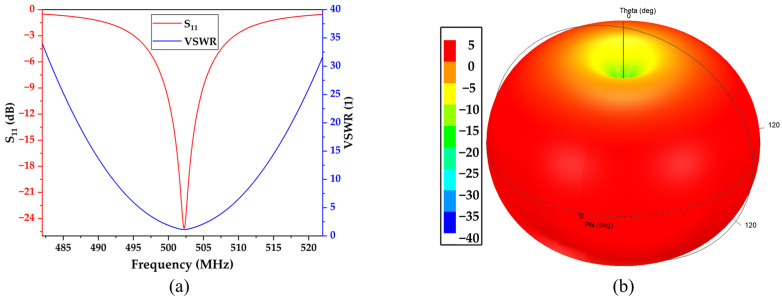
(**a**) Simulated *S*_11_ and VSWR of helix antenna; (**b**) the normalized radiation pattern of the antenna.

Figure 9 shows the simulated *S*_11_ and Voltage Standing Wave Ratio (VSWR) of the antenna, which are −25.13 dB and 1.12, respectively, effectively meeting the requirements of the communication system. From the graph, the resonant frequency of the antenna is 502.24 MHz, which is very close to the resonant frequency of the SAW resonator. Additionally, the −10 dB bandwidth of the antenna is 4.7 MHz, ranging from 499.89 MHz to 504.59 MHz, which covers the range of resonant frequency variations within the temperature measurement range of the SAW resonator. This indicates that the dimensional design of the antenna is reasonable.

**Figure 10:**
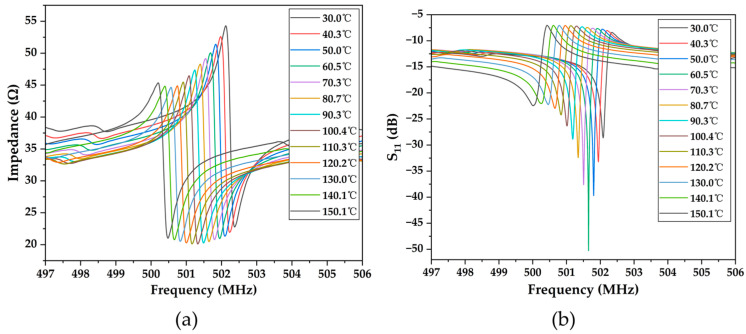
Impedance curve (**a**) and return loss *S*_11_ curve (**b**) of the SAW sensor at different temperatures after matching.

Due to the mismatch in impedance between the SAW resonator and the antenna, an impedance-matching network must be added to improve the energy transfer between the two, as depicted in Figure 7c. Figure 10 displays the impedance curve and *S*_11_ curve of the SAW sensor measured through a VNA at various temperatures after the addition of the impedance matching network. It is evident from the graph that the impedance of the SAW sensor at the resonance point decreases in magnitude with increasing temperature, while the deviation from the 50 Ω standard load first decreases and then increases. It measures 53.78 Ω at 30 °C and 49.70 Ω at 60 °C, which is closest to the ideal 50 Ω. As the temperature continues to rise, the impedance further decreases and deviates from 50 Ω, reaching 43.69 Ω at 150 °C. Correspondingly, with increasing temperature, the *S*_11_ parameter of the SAW sensor first increases and then decreases. It reaches a minimum at 30 °C (−28.63 dB) and a maximum at 60 °C (−50.27 dB), then decreases again to −22.96 dB at 150 °C. However, even at its minimum value, there is a significant improvement in the *S*_11_ parameter of the SAW resonator compared to before matching, demonstrating the effectiveness of the matching. Similarly to before matching, the resonant frequency of the SAW sensor still decreases with increasing temperature after matching. In addition, at the same temperature after matching, the resonant frequency of the SAW sensor decreases slightly compared to before matching, which is a normal phenomenon after the addition of the impedance matching circuit.

**Figure 11:**
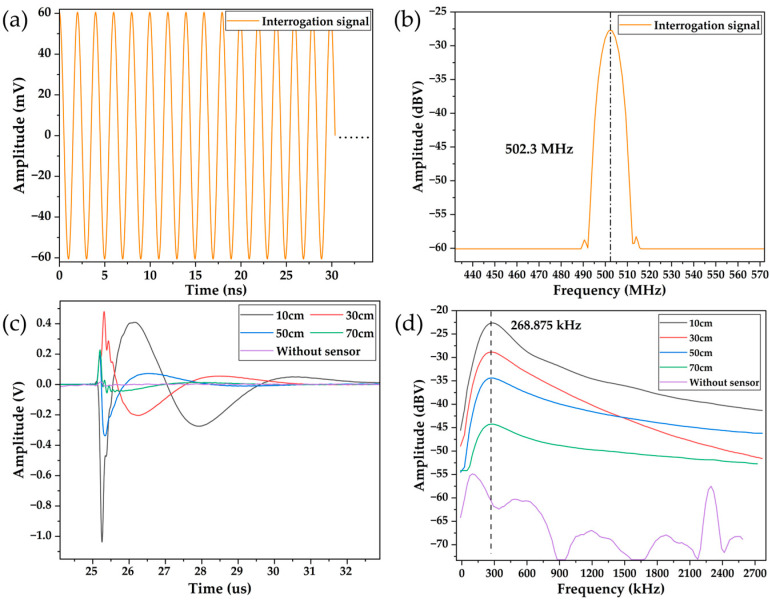
(**a**) Interrogation signal in the time domain; (**b**) interrogation signal in the frequency domain; (**c**) echo signal at different distances in the time domain; and (**d**) echo signal at different distances in the frequency domain.

After the impedance matching is complete, the antenna is soldered to the PCB board to form the wireless SAW temperature sensor, as shown in Figure 7d. Figure 11a,b show the time domain and frequency domain plots of the interrogation signal emitted by the mother antenna. The amplitude of the interrogation signal is approximately 60 mV with a frequency of 502.3 MHz, corresponding to the resonant frequency of the SAW sensor at room temperature. The switching period of the RF switch in the transceiver is 50 us. To ensure full resonator oscillation, the transceiver continuously transmits the interrogation signal for 25 us before switching to the receive mode. To capture the complete signal, the receive mode also lasts for 25 us. The echo signal can be observed on the oscilloscope by placing the wireless SAW sensor close to the mother antenna within a certain range. Figure 11c,d display the time domain and frequency domain plots of the processed echo signal obtained when the wireless SAW temperature sensor is placed at different distances from the mother antenna at room temperature. Figure 11c illustrates that as the distance between the SAW sensor and the mother antenna increases, the amplitude of the echo signal decreases continuously. At 10 cm, the peak-to-peak value of the echo signal is around 1.5 V, while at 70 cm, it drops to around 200 mV. In addition, closer distances result in stronger signal intensity and longer signal duration. At 10 cm, the signal lasts for nearly 10 μs after the transmission signal stops, indicating prolonged SAW oscillation. Figure 11d also indicates that the amplitude of the frequency domain signal decreases with increasing distance between the SAW sensor and the radiator, dropping from −22 dBV at 10 cm to −45 dBV at 70 cm. However, compared to the noisy waveform in the absence of the sensor, the signal peak is still quite prominent. It can be observed that the frequency of the echo signal remains unchanged with the distance, remaining constant at 268.875 kHz.

The wireless temperature measurement experiment was conducted at 15 cm from the mother antenna within the temperature range of 30 °C to 150 °C. It is noteworthy that the system circuit contains a mixer that shifts the frequency of the high-frequency echo signal to a lower frequency. Therefore, the final frequency *f* of the processed echo signal can be calculated using the following formula:

(4) $$f=f_{t}-f_{0}$$

where *f_t_* is the frequency of the interrogation signal and *f*_0_ is the frequency of the initial echo signal at the mother antenna returning from the SAW sensor. During the temperature measurement experiment, *f_t_* remains constant, and it can be seen from Figure 8b that *f*_0_ decreases with an increase in temperature, resulting in an increase in *f* with rising temperature, which has an opposite trend to *f*_0_.

**Figure 12:**
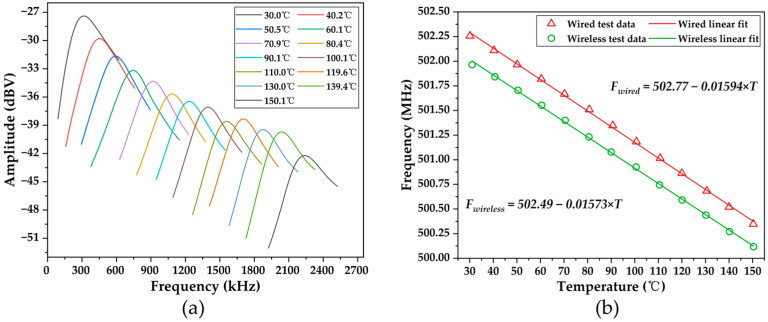
(**a**) Echo signal in the frequency domain at different temperatures; (**b**) comparison of the frequency and temperature relationship between the wired test and wireless test.

Figure 12a displays the frequency domain plots of the echo signal at different temperatures. It can be observed from the graph that the frequency of the echo signal increases continuously with increasing temperature, ranging from 335.75 kHz at 31 °C to 2182 kHz at 150.4 °C. Simultaneously, the amplitude of the echo signal decreases continuously. This phenomenon occurs because the resonant frequency of the SAW resonator gradually decreases with increasing temperature while the frequency of the interrogation signal remains constant. Consequently, at higher temperatures, the mismatch between the frequency of the interrogation signal and the resonant frequency of the SAW resonator leads to the incomplete excitation of the SAW resonator, resulting in the attenuation of the echo signal.

Future research will focus on modifying *f_t_* during the temperature measurement to improve the signal strength and measurement range without affecting the results. Additionally, the performance of the SAW resonator deteriorates with increasing temperature, and changes in the impedance of the SAW sensor lead to imperfect matching, which also contributes to the reduction in the amplitude of the echo signal.

The temperature–frequency relationship of the echo signal was extracted from Figure 12a, and Formula (2) was employed to deduce the temperature–frequency relationship of the SAW temperature sensor. The fitted results were compared with those obtained from the wired testing, as depicted in Figure 12b. It was observed that the resonant frequency for wireless testing was slightly lower compared to wired testing at the same temperature. This was due to the presence of the impedance-matching circuit and antenna coupling. The calculated wireless temperature sensitivity and TCF were −15.73 kHz/°C and −31.34 ppm/°C, respectively. These values closely matched those of −15.89 kHz/°C and −31.63 ppm/°C obtained from the wired testing. Furthermore, the fitted results exhibited excellent linearity, with a linear coefficient of 0.99926, which was consistent with the wired testing. These findings indicate that the constructed wireless temperature sensor does not compromise the temperature measurement capability of the SAW resonator, thus demonstrating its efficacy in temperature detection.

## 4. Conclusions

This article presents a wireless temperature sensor based on an AlN SAW resonator with a frequency of 502.3 MHz. The resonant modes, *S*_11_ curve, and temperature–frequency characteristics of the designed SAW resonator were simulated in COMSOL 6.0. The fabricated SAW resonator was then electrically characterized, and the measured *S*_11_ parameters closely matched the simulation results. The temperature–frequency characteristics of the SAW resonator were then investigated, revealing a measured wired temperature sensitivity of −15.89 kHz and a TCF value of −31.63 ppm/°C. Next, a helix antenna with a resonant frequency identical to that of the SAW resonator was designed using AnsysEM21.2 HFSS. An impedance matching network was added between the SAW resonator and the helix antenna due to impedance mismatch. The performance of the SAW sensor after adding the impedance-matching network was studied. The matched *S*_11_ parameters fluctuated with temperature but exhibited significant improvement compared to the unmatched conditions. 

After assembling the wireless SAW temperature sensor, wireless signal tests were conducted at various distances between the SAW sensor and the parent antenna. It was observed that the signal remained distinguishable even at 70 cm, and the frequency of the echo signal did not change with the distance. Wireless temperature measurement experiments showed a steady increase in the frequency of the echo signal with rising temperature. After fitting the temperature–frequency relationship, the wireless temperature sensitivity and TCF were calculated to be −15.73 kHz and −31.34 ppm/°C, respectively. These results closely align with those obtained from the wired testing of the SAW resonator, indicating the practicality of the designed wireless temperature sensor.

## Figures and Tables

**Table 1:** Material constants used in the simulation [38].

		AlN		Si	Mo
Elastic constants *c_ij_* (GPa)	*c*_11_	410.06	Young’s modulus *E* (GPa)	170	385
	*c*_12_	100.69			
	*c*_13_	83.82			
	*c*_33_	286.24			
	*c*_44_	100.58			
	*c*_66_	154.70			
Temperature coefficient of constants *TEC_ij_* (1 × 10^−6^∙K)	*TEC*_11_	−10.65	Temperature coefficient of Young’s modulus *TCE* (1 × 10^−6^∙K)	−63	−181
	*TEC*_12_	−11.67			
	*TEC*_13_	−11.22			
	*TEC*_33_	−11.13			
	*TEC*_44_	−10.82			
	*TEC*_66_	−10.80			
Thermal expansion *α_ij_* (1 × 10^−6^∙K)	*α*_11_	5.27		2.6	3.49
	*α*_22_	5.27		2.6	3.49
	*α*_33_	4.15		2.6	3.49
Mass density ρ (kg/m^3^)	ρ	3300		2329	10,200

**Table 2:** Parameters of the designed helix antenna.

*D* (Helix Diameter)	*S* (Space)	*N* (Number of Turns)	*d* (Wire Diameter)
5.8 mm	2 mm	21.5	1.2 mm

## Data Availability

Data is contained within the article.
